# Monotony in the consumption of fruits and vegetables and food environment characteristics

**DOI:** 10.11606/S1518-8787.2019053000705

**Published:** 2019-08-23

**Authors:** Raquel de Deus Mendonça, Mariana Souza Lopes, Patrícia Pinheiro Freitas, Suellen Fabiane Campos, Mariana Carvalho de Menezes, Aline Cristine Souza Lopes

**Affiliations:** I Universidade Federal de Minas Gerais. Escola de Enfermagem. Departamento de Nutrição. Programa de Pós-Graduação em Enfermagem. Grupo de Pesquisas de Intervenções em Nutrição. Belo Horizonte, MG, Brasil; II Universidade Federal de Minas Gerais. Faculdade de Medicina. Departamento de Medicina Preventiva e Social. Programa de Pós-Graduação em Saúde Pública. Grupo de Pesquisas de Intervenções em Nutrição. Belo Horizonte, MG, Brasil; III Secretaria Municipal de Saúde. Núcleo de Apoio a Saúde da Família. Belo Horizonte, MG, Brasil; IV Fundação Oswaldo Cruz. Escola Nacional de Saúde Pública. Programa de Pós-Graduação de Epidemiologia em Saúde Pública. Grupo de Pesquisas de Intervenções em Nutrição. Rio de Janeiro, RJ, Brasil

**Keywords:** Food Intake, Vegetables, Fruit, Diet Surveys, Food and Nutrition Education

## Abstract

**OBJECTIVE:**

To analyze the quantity and diversity in the consumption fruits and vegetables, as well as its relationship with the consumer’s purchase characteristics and food environment.

**METHODS:**

Baseline study stemming from a controlled and randomized community trial investigating a sample representative of Primary Health Care services (Health Academy Program) of Belo Horizonte, state of Minas Gerais. The intake of fruits and vegetables was analyzed in servings/day, whereas diversity was assessed by the Food Frequency Questionnaire. Users were also questioned on the frequency, purchase location and availability of these foods at their households. To assess the consumer’s food environment, commercial establishments within a 1.6 km radius around the program unit sampled were audited.

**RESULTS:**

3,414 adults and older adults (88.1% women) were investigated, as well as 336 commercial establishments, in 18 units of the Health Academy Program. The average consumption of fruits and vegetables was adequate [5.4 (SD = 2.1) servings/day] but monotonous, with average daily intake of two different types. In the establishments audited, a good diversity (77.7% and 85.0%) and variety (74.5% and 81.4%) of fruits and vegetables was observed, although with lower quality of vegetables (60.4%). After adjusting for sociodemographic variables, we identified that knowledge on food crops (p = 0.006), increased monthly availability of fruits at households (p < 0.001), and greater variety of fruits (p = 0.03) and quality of vegetables (p = 0.05) in commercial establishments could improve the quantitative intake of fruits and vegetables, whereas a greater variety of fruits (p = 0.008) would increase consumption diversity.

**CONCLUSIONS:**

The intake of fruits and vegetables was quantitatively adequate but monotonous, being influences by the consumer environment. Such results highlight the need for improving educational actions in health services and programs, in addition to acting on the consumer environment, aiming to promote and maintain the adequate and diversified consumption, as recommended by Brazilian guidelines for proper and healthy eating.

## INTRODUCTION

Over the centuries, mankind has developed technologies to improve the availability and shelf-life of foods. An important step in that direction was the industrialization^1^ , which in a short time allowed numerous countries to be dominated by food processing^2^ .

As a result of industrialization, the intake of fresh foods decreased^2^ , including fruits and vegetables (F&V)^3 , 4^ . The inadequate intake of F&V is one of the ten central factors in the determination of the global burden of diseases and is characterized by the insufficient quantity or lack of variety, with consequent reduction of the food repertoire and low intake of nutrients^5^ .

The inadequate intake of F&V is globally widespread and often investigated by studies and population surveys in several countries^3 , 4 , 6^ . However, none of them evaluated the diversity of F&V consumed. In this context, even if the strategies aimed at expanding the access and intake of F&V are the priority of health policies around the world^5^ , they are limited to stimulating and assessing the intake according to recommendations (five servings/day). Overall, they do not consider the importance of diversity in food and little cover the role of food environment on food intake, especially in collective contexts such as health services^10 , 11^ and in developing countries.

Environment can favor both unhealthy as healthy behaviors due to its strong interference on purchasing decisions and choices of individuals^10^ . Studies show that a greater variety and availability of food in the consumer’s environment can favor their intake^13 , 14^ . A review study conducted in the United States pointed out that the low availability of F&V was associated with a worse diet quality^14^ .

Given the above, this article aimed to analyze the quantity and diversity of F&V intake and their relations with the consumer’s acquisition characteristics and food environment.

## METHODS

This study analyzed the baseline (2013/2014) of a controlled and randomized community trial in a probabilistic and representative sample of the Health Academy Program (PAS) units of Belo Horizonte, state of Minas Gerais. The research included PAS users and the food environment in their surroundings. Other research details can be seen in Costa et al.^10^ and Menezes et al.^15^

The PAS was chosen as research scenario because this is a Primary Care point that aims to contribute to the construction of health-promoting environments. To do so, it has infrastructure, equipment and qualified staff for the guidance toward healthy ways of life, including the promotion of proper and healthy nutrition, and for food and nutritional security actions^16 , 17^ . In the municipality investigated, the PAS offers oriented physical exercise practices and health promotion actions in partnership with the Nucleus of Support to Family Health (NASF)^17^ .

The study was conducted within the standards research by the Declaration of Helsinki and approved by the Research Ethics Committees of the Federal University of Minas Gerais (0537.0.0203.000-11) and the City Hall of Belo Horizonte (0537.0.0203.410-11A). In addition, the study was registered in the Brazilian Registry of Clinical Trials (RBR-9h7ckx), according to the criteria required by the International Committee of Medical Journal Editors (ICMJE) and the World Health Organization (WHO).

### Sampling of PAS Units Participating in the Study

In the sampling process, 42 of the 50 PAS units installed in the municipality were considered. Six units located in areas of low health vulnerability were excluded due to their reduced number in the municipality, and two others were excluded due to the intense performance of intervention studies. Thus, 18 (42.8%) units distributed through the nine regions of the municipality^10^ were randomly assigned, representing the total with a confidence level of 95% and 1.4% error. After data collection, in order to verify the maintenance of sample representativeness, statistical analysis were carried out, comparing the sociodemographic data of all units (n = 50) with those of the sample (n = 18), with the same levels of confidence and error.

In the sampled units, all participants were invited to participate in the study (n = 3,763). Of these, 3,414 (90.7%) were interviewed, and one accounted for 237 refusals (6.3%) and 112 exclusions (3.0%)^15^ . Inclusion criteria were: being at least 20 years old and having regularly participated in PAS activities in the month prior to the beginning of data collection. Exclusion criteria were: being pregnant and having cognitive impairment that hindered questionnaire application. Data of participation in the activities were collected through the daily frequency record held by the service, whereas cognitive impairment was observed from the initial assessment of the user in their entrance into PAS and from the evaluation conducted by the research team^15 , 17^ .

To characterize the food environment, establishments that traded F&V and free fairs within a *buffer* with 1,600-meter radius around each PAS unit sampled were investigated. Such distance was chosen because it is adequate for carrying out purchases without the need for motorized transportation. Data of establishments and free fairs were made available by the municipality and added to those found spontaneously in the territory of PAS units during the visits to registered establishments^10^ .

Data collection was conducted by Nutrition academics and health professionals semiannually trained for using instruments and performing interviews, under the guidance of the field supervisor and the main researcher. Data were subjected to consistency analysis before and after tabulation.

### Individual Evaluation

Information was obtained from individuals through a questionnaire submitted to pilot study and applied in person by trained interviewers. The questionnaire was built from national studies and previous experience of the research group^8 , 15 , 18 , 19^ and included sociodemographic and economic characteristics, health aspects, acquisition profile, nutritional status and intake of F&V.

To register the intake of F&V, the Brief Evaluation Questionnaire on Fruit and Vegetable Consumption (QBrief-F&V) was used, validated for the study population^19^ , which investigated the frequency (1–2 days/week, 3–4 days/week, 5–6 days/week, daily – including Saturdays and Sundays; almost never/never) and the number of servings consumed. To verify the diversity of consumption, the specific Questionnaire of Food Frequency (QFF) was applied regarding the last six months, including 14 fruits and 22 vegetables. Diversity was assessed by the daily intake of the F&V analyzed^19^ . To characterize the F&V acquisition profile, variables related to knowledge on the concept of food crop and the availability of F&V at home were used, in addition to the frequency and type of establishment chosen for the purchase of F&V^15^ .

Sociodemographic and economic characteristics investigated were sex, age, education level (years), marital status (common-law marriage, divorced, widowed or single) and occupation (housewife, retired, employed or unemployed). Material goods within the residence and education level of the family head were used for the economic classification of participants into five classes, from A (the richest) to E (the poorest), as determined by the Brazilian Economic Classification Criterion^20^ .

Health aspects were assessed by the self-reported medical diagnosis of diabetes, hypertension and dyslipidemia, as well as by health perception (very good, good, regular, bad or very bad). In addition, information on the frequency (days) and duration (minutes) of physical exercise practice and on the user’s time of participation in PAS (difference between the dates of data collection and entry of the user in the program) were collected.

For the assessment of nutritional status, the anthropometric measurements of weight and height were used, based on which the body mass index (BMI) was calculated, classified according to the criteria defined by WHO^21^ . Weight was assessed by a single measuring in a digital scale of the brand Marte^®^, with a 180 kg capacity and 100 g precision. The height was also assessed by a single measuring in a portable single, Alturexata^®^ brand, with capacity for 220 cm, adopting the techniques recommended by the Ministry of Health^22^ .

### Evaluation of Food Environment

Visits were made to convenience stores, municipal market, grocery stores of the private sector and subsidized by the city hall, local or neighborhood markets, supermarkets of large networks, hypermarkets, whole retail stores and bakeries^23^ . The following characteristics of the consumer environment were verified for F&V: diversity, variety and quality. To this end, instruments developed and validated for the Brazilian context^24^ were used, which were adapted including the 10 most purchased types of fruits and vegetables in Belo Horizonte (except canned or frozen fruits, and tubers and roots), according to the 2008–2009 Family Budgets Survey^8^ .

Diversity was assessed by the number of F&V available in the establishment, whereas variety was verified by the number of different types of a same species (green apples, Fuji apple and Gala apple, for instance). Quality was classified as good or bad from a subjective evaluation, considering the presence of blemishes and bruises or the appearance of “rotten” or “too ripe”^24^ .

### Data Analysis

The intake of F&V was analyzed in quintiles of daily servings consumed and described in an aggregate manner according to the PAS units investigated. Characteristics of the food environment, in turn, were analyzed according to the PAS units.

To verify the association between participants’ characteristics and quintiles of F&V continuous, the Chi-square statistical test of linear trend was used for categorical variables and analysis of variance (Anova) for continuous variables. The multilevel linear regression was used to assess whether the indicators of food acquisition profile (individual level) and consumer food environment (diversity, variety and quality of fruits and vegetables, totaling six variables) explained the variations in the quantitative (servings/day) and quality intake (daily diversity) of F&V. Such analysis allows one to consider the hierarchical structure of the data aggregation level, in addition to the insertion of individual and contextual variables. Thus, the individual consumption (daily servings and diversity) of F&V consisted of the two outcomes analyzed, with the participants (level 1) nested according to PAS units (level 2).

In addition to the food acquisition profile at an individual level, sociodemographic variables were added for model adjustment. The construction of the model occurred in a hierarchical manner, first building the null model (with including solely of the random intercept), followed by the insertion of individual variables and, finally, environment variables. Final models showed the variables that presented higher association (small p value and higher percentage of variability explained) with the outcomes F&V servings and diversity consumed, after adjusting for individual variables. It should be highlighted these are models with random intercept, which do not show this pattern for coefficients (of fixed effects).

The intraclass correlation coefficient (ICC) was quantified to analyze the variability within and between territories, providing the ratio of total variability, which depends on the differences between territories. The percentage of proportional variation in variance (PVP) was calculated between the null and the final models, aiming to analyze the extent to which the covariates explained the variation in outcomes between territories.

All variables were analyzed in the program Stata/SE version 14.0 (Stata Corp., College Station, TX, USA), and the statistical significance was established in 5%.

## RESULTS

3,414 individuals were interviewed and 336 commercial establishments in the territories of 18 PAS units were audited. Most participants were women (88.1%) and middle-aged adults [56.7 (SD = 11.7) years old], with reported average consumption of 5.4 (SD = 2.1) servings of F&V of two different types per day ( Table 1 ).

**Table 1:** Characteristics of the participants according to the quintile of fruits and vegetables intake. Belo Horizonte, state of Minas Gerais, 2013–2014.

Variable	Total	Quintile of total consumption of fruits and vegetables					p*

		Q1	Q2	Q3	Q4	Q5	
Fruits and vegetables (servings/day)	5.4 (2.1)	3.0 (0.6)	4.3 (0.3)	5.2 (0.2)	6.2 (0.3)	8.5 (1.9)	< 0.001
Age (years)	56.7 (11.7)	54.7 (12.6)	56.2 (11.8)	56.8 (11.3)	57.7 (11.3)	58.6 (11.1)	< 0.001
Sex (%)							0.001
Female	88.1	89.5	90.1	88.0	86.9	84.5	
Male	11.9	10.5	9.9	11.0	13.1	15.5	
Years of study	7.2 (4.1)	7.1 (4.2)	7.1 (4.0)	7.5 (4.1)	7.2 (4.0)	7.3 (4.2)	0.39
Marital status (%)							0.71
Common-law marriage	61.6	60.8	62.9	62.5	61.0	61.0	
Divorced	8.3	8.2	8.3	7.9	7.8	9.2	
Single	14.1	15.9	13.6	13.1	13.9	13.7	
Widowed	16.0	15.1	15.2	16.5	17.3	16.1	
Occupation (%)							0.02
Housewife	28.7	31.1	27.8	29.3	29.6	25.5	
Retiree	36.7	30.4	33.9	36.3	40.4	43.8	
Unemployed	2.0	2.9	1.9	2.1	1.8	1.2	
Employee	32.6	35.6	36.4	32.4	28.3	29.5	
Economic class (%)							0.83
A/B	29.5	28.7	30.0	30.1	29.7	29.2	
C	55.0	53.3	55.6	57.3	56.0	53.1	
D/E	15.5	18.0	14.4	12.6	14.3	17.7	
PAS time (months)	19.8 (15.3)	18.8 (15.1)	19.5 (14.7)	20.0 (15.7)	20.5 (15.7)	20.4 (15.3)	0.21
BMI (kg/m^2^)	27.9 (4.8)	27.6 (4.8)	27.6 (4.8)	28.2 (4.7)	27.9 (5.0)	28.0 (5.1)	0.06
Smoking habit (%)	5.7	6.1	4.3	7.2	5.2	5.6	0.99
Chronic diseases (%)							
Diabetes	16.9	12.5	13.9	18.3	19.9	21.0	< 0.001
Arterial hypertension	53.2	47.8	51.8	54.4	57.1	55.9	< 0.001
Dyslipidemia	44.1	41.2	42.5	46.1	46.5	44.8	0.62
Health perception (%)							0.48
Very good/good	71.7	71.4	73.4	70.7	73.7	69.6	
Regular/Poor/Very poor	28.3	28.6	26.6	29.3	26.3	30.4	
Practice of physical exercises (minutes/week)	209.6 (79.9)	201.9 (63.4)	206.8 (71.7)	208.6 (86.9)	218.3 (89.8)	213.0 (84.8)	0.004
Daily diversity (types)							
Fruits	1.5 (1.4)	0.8 (1.0)	1.2 (1.3)	1.6 (1.4)	1.7 (1.4)	1.9 (1.5)	< 0.001
Vegetables	0.9 (1.4)	0.7 (1.2)	0.8 (1.3)	0.9 (1.4)	0.9 (1.2)	1.1 (1.6)	< 0.001
Fruits and vegetables	2.3 (2.1)	1.6 (1.7)	2.1 (2.0)	2.6 (2.3)	2.6 (2.2)	3.1 (2.4)	< 0.001

PAS: Health Academy Program; BMI: body mass index

Values presented as % or mean (standard deviation).

* Linear trend Chi-square test (categorical variables) and analysis of variance (Anova; continuous variables), according to the quintile of consumption of fruits and vegetables.

Participants in the last quintile of F&V intake [8.5 (SD = 1.9) servings/day], in comparison with those in the first quintile [3.0 (SD = 0.6) servings/day], were older, with a higher proportion of retirees, carriers of some chronic disease and more physically active individuals. Women reported a high consumption of F&V, which decreased as the F&V quintiles increased, a situation opposite to that of men ( Table 1 ).

Individuals in the last quintile of F&V consumption reported eating greater diversity of these foods than those in the first quintile ( Table 1 ). The main fruits consumed were banana, orange and apple, whereas vegetables were tomato, lettuce and carrot ( Figure ). People with higher consumption of F&V reported greater knowledge on food crops, conducted purchases more often (approximately once a week) and had a higher availability of F&V at home ( Table 2 ).

**Figure:**
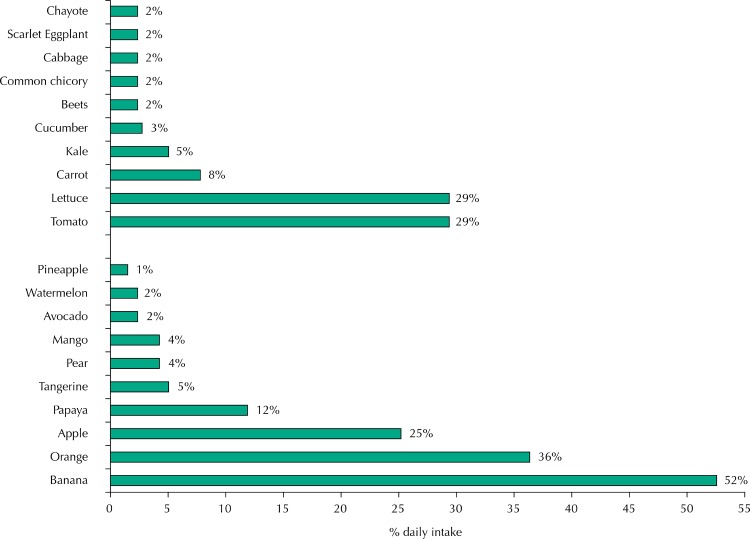
Main fruits and vegetables consumed daily. Belo Horizonte, state of Minas Gerais, 2013–2014.

**Table 2:** Food acquisition profile according to the quintile of fruits and vegetables intake. Belo Horizonte, state of Minas Gerais, 2013–2014.

Variable	Total	Quintile of total consumption of fruits and vegetables					p*

		1	2	3	4	5	
Knowledge on harvests (%)	75.0	70.8	71.7	78.7	76.4	77.9	< 0.001
Establishment where fruits and vegetables are bought (%)							
Supermarkets	40.5	41.6	39.4	39.7	41.3	40.4	0.91
Subsidized Grocery Store	40.8	38.6	39.1	43.5	39.2	43.7	0.07
Private Grocery Store	80.5	82.0	80.9	80.0	81.1	78.5	0.14
Free Trade Fairs	81.2	80.0	82.1	82.4	80.6	81.0	0.89
Municipal Market	23.7	25.5	23.0	23.5	25.3	21.4	0.23
Vegetable garden or donation	22.8	22.1	22.9	21.2	24.7	23.5	0.37
Fruit acquisition frequency (%)							< 0.001
3 times or more/week	8.6	6.5	8.4	8.4	8.8	11.3	
1–2 times/week	77.9	73.8	79.0	76.6	80.7	79.7	
3 times or less/month	13.5	19.7	12.6	15.0	10.5	9.0	
Vegetables acquisition frequency (%)							< 0.001
3 times or more/week	12.9	10.6	12.3	11.9	12.9	16.8	
1 to 2 times/week	76.1	73.1	76.9	76.9	77.9	76.0	
3 times or less/month	11.0	16.3	10.8	11.2	9.2	7.2	
Monthly Availability (days)							
Fruits	27.1 (6.6)	25.5 (8.3)	27.1 (6.5)	27.2 (6.3)	28.2 (5.3)	27.9 (5.8)	< 0.001
Vegetables	28.1 (5.5)	27.4 (6.7)	27.9 (5.7)	28.1 (5.6)	29.0 (4.0)	28.6 (5.0)	< 0.001

Values presented as % or mean (standard deviation).

* Linear trend Chi-square test (categorical variables) and analysis of variance (Anova; continuous variables), according to the quintile of consumption of fruits and vegetables.

The characteristics of the consumer’s food environment according to PAS units investigated are presented in Table 3 . Most presented adequate variety and diversity, with percentages above 70%; however, the quality was inferior (< 60%).

**Table 3:** Consumption of fruits and vegetables and consumer food environment in the Health Academy Program. Belo Horizonte, state of Minas Gerais, 2013–2014.

Region	PAS unit	Adequacy (%)					

		Variety of fruits	Variety of vegetables	Fruit diversity	Vegetables diversity	Fruit quality	Vegetables quality
1	1	70.0	70.0	70.0	80.0	85.7	87.5
	2	90.0	90.0	90.0	90.0	100.0	77.8
2	3	77.1	79.2	77.1	72.2	94.1	90.9
	4	83.3	83.3	91.7	75.0	90.0	100.0
3	5	63.6	81.8	75.8	93.9	54.1	38.7
	6	68.7	80.6	80.6	93.5	65.2	37.9
4	7	70.6	88.2	76.5	88.5	54.5	33.3
	8	43.7	62.5	50.0	88.2	73.3	42.8
5	9	83.3	83.3	83.3	68.5	73.3	56.2
	10	70.0	70.0	70.0	83.3	57.1	25.0
6	11	81.8	63.6	81.8	70.0	71.4	50.0
	12	77.8	55.6	77.8	81.8	60.0	62.5
7	13	88.2	100.0	88.2	77.8	80.0	70.6
	14	81.2	95.4	90.9	100.0	78.9	77.2
8	15	70.0	90.0	70.0	95.4	50.0	22.2
	16	71.4	73.3	80.0	90.0	81.2	33.3
9	17	87.1	80.6	87.1	80.0	96.0	82.1
	18	87.7	81.2	87.5	87.5	84.6	73.3

Total		75.4	80.6	79.8	84.2	75.5	60.4

PAS: Health Academy Program; F&V: fruits and vegetables

Variety: number of different types of F&V of the same species in the establishment.

Diversity: number of F&V available at the establishment.

In the multilevel analysis, after adjusting for sociodemographic variables, one could see that knowledge on crops and the increased monthly availability at home and frequency of fruit purchases could increase consumption (servings/day) of F&V ( Table 4 ). Regarding environment, a higher consumption (servings/day) was associated with commercial establishments with a greater variety of fruits and quality of vegetables, regardless of individual variables. Intragroup variance and ICC have reduced progressively until the final model presented (null model σ^2^ = 2.04, ICC = 0.011; model level 1: σ^2^ = 2.02, ICC = 0.009; model levels 1 and 2: σ^2^ = 2.01, ICC = 0.004), indicating the covariates inserted helped explaining the F&V intake. In line with this result, the PVP calculation showed the model covariates explained 34.7% of the variable quantitative consumption of F&V.

**Table 4:** Multilevel linear regression models for the intake of fruits and vegetables, with both quantitative (servings) and qualitative (consumption variety) measures as outcome variables. Belo Horizonte, state of Minas Gerais, 2013–2014.

Explanatory variables	B	Standard error	95%CI	p
Model with the outcome variable fruit and vegetables intake (servings)				

Individual variables – level 1				
Knowledge on harvests^a^	0.27	0.09	0.08–0.46	0.006
Buys fruits 3 times or more/week	0.58	0.18	0.22–0.94	0.002
Buys fruits 1 to 2 times/week	0.33	0.13	0.07–0.58	0.012
Monthly Availability of Fruit (days)	0.03	0.01	0.02–0.05	< 0.001
Adjustment variables				
Age (years)	0.02	0.00	0.01–0.02	< 0.001
Sex	0.52	0.13	0.27–0.77	< 0.001
Years of study	0.01	0.01	–0.01–0.03	0.56
Environment variables – level 2				
Variety of fruits	1.07	0.48	0.13–2.01	0.03
Vegetables quality^b^	1.59	0.83	–0.03–3.21	0.05
Random effect				
Variance between groups (τ00)	0.14	0.06	0.06–0.33	-
Intra-group variance (σ^2^)	2.02	0.03	1.96–2.07	-

Model with the outcome variable diversity in the consumption of fruits and vegetables				

Individual variables – level 1				
Adjustment variables				
Age (years)	0.00	0.00	–0.01–0.01	0.58
Sex	0.05	0.12	–0.20–0.30	0.70
Years of study	0.00	0.01	–0.02–0.02	0.79
Environment variables – level 2				
Fruit diversity	0.27	0.10	0.07–0.46	0.008
Vegetables quality^b^	0.32	020	0.07–0.70	0.11
Random effect				
Variance between groups (τ00)	0.31	0.08	0.19–0.53	-
Intra-group variance (σ^2^)	2.02	0.03	1.97–2.08	-

B: Coefficient B

^a^ Knows food crops (yes or no).

^b^ Quality of vegetables (bad or good).

The variables of food acquisition profile investigated were not associated with the diversity of F&V consumed; however, the variables of territory offer had a positive association with it. Covariates inserted in the model helped explaining 28.3% of variety of F&V consumption.

## DISCUSSION

Participants in this program of health promotion, part of the Brazilian primary care, reported an adequate consumption of F&V (daily average of five servings). However, when evaluating the consumption qualitatively, food monotony was observed, with an average of only two different types of F&V consumed per day. The F&V acquisition profile interfered in consumption, with emphasis on the knowledge on food crops and purchase frequency and home availability of fruits. The consumer’s food environment, in turn, proved to be important for both the quantitative (servings/day) as the qualitative intake (consumption diversity).

Such amount was surprisingly superior to national and international values^3 , 4 , 6 , 7^ . In England (2011/2012), 70% of adults and 59% of older people did not consume the recommended amount of F&V^3^ . In the United States, the average daily consumption of such foods is 2.7 servings per day^4^ . In Latin America, in countries such as Argentina (2013) and Chile (2009/2010), 5.0% and 84.3% of the population did not present adequate intake of F&V, respectively^6 , 7^ . Research conducted in Brazil (from 1987 to 2009) show decline in average quantities purchased^25^ and F&V consumption^26^ , estimating that less than 10.0% of the population presented adequate intake^8^ .

The intake found in this study was also higher than that identified in other study carried out in the PAS. When analyzing those entering a triennium (2008 to 2010) on PAS, Costa et al.^27^ verified 75.3% inadequacy in the F&V consumption. Part of the difference in results can be attributed to the time of user participation in the health service; in this study, the average was 20 months. Participation in health promotion service, although focused on the practice of physical exercises, seems to increase the adoption of other healthy habits of living, such as healthy foods, in addition to increasing all knowledge regarding the foods crop^16^ .

In this study, therefore, we observed the potential of the health promotion service in reversing the quantitative deterioration of the F&V consumption. Such result reveals as possible path for governments to promote positive changes in this respect. However, it also points out the challenge of food monotony and the imminent need for discussing it in the context of educational actions and recommendations, rescuing and clarifying the WHO message that appropriate consumption consists of the regular, diversified and quantitatively recommended intake of F&V^5^ .

There are more than 200 types of fruits and vegetables within different cultures, which differ by caloric density, water content and diversity of micro-nutrients, resulting in benefits to health and protection against diseases^5 , 28^ . Insufficient food diversity can hinder the maintenance of healthy eating habits in the long term, resulting in the reduction of the population food repertoire and in less nutritional content of food.

Low diversity in F&V intake can also be related to the lowest price, time and complexity required for food preparation, as well as an attempt to overcome obstacles such as the low quality of F&V. These issues become even more relevant when one considers that vulnerable areas were studied, areas with an average income that is below the Brazilian population^23 , 29^ .

A prior study on commercial establishments in the PAS territory of Belo Horizonte showed that, in some units, there was a limited access to establishments with proper F&V availability and variety^10^ . The food environment should support healthy choices, offering ample choices of accessible, varied and quality foods. This study reinforces such information by suggesting that variables of the consumer’s food environment (variety and quality) were important, in addition to matters related to F&V purchase. In agreement with the results, in a study performed in Brazil, the reports of women on food acquisition revealed the variety of F&V in the establishments had an impact on the purchase of healthy foods at the expense of ultra-processed food^30^ .

Our results emphasize the importance of promoting adequate, diverse and healthy food in primary health care, considering its potential to incentivize healthy ways of living. However, we must move beyond the quantitative adequacy and associated factors, studying also the diversity consumed^5 , 28^ . In addition, still little is known on the influence of consumer’s environment in eating, a situation which shows how the food is still studied without considering the complexity in which it is immersed.

It is suggested that further studies are conducted to better understand the relationship between intake, monotony and food environment (supply and demand), aimed at addressing questions such as: does the restricted environment leads to food monotony or is food monotony (little diversified demand) the one that leads to the precarious food supply in the territory?

These results, albeit unprecedented and relevant, require caution regarding external validity. Participants are regulars of a service geared towards health promotion, focusing on the practice of physical exercise and, therefore, more aware regarding their health, what sets them apart from the general population^9^ . Nonetheless, the results show the importance of working in other research scenarios, as well as of understanding the actions provided in health services as possible paths to cope with the challenges faced by the sector. The difficulty in obtaining reliable measures of food consumption has been singled out as an important limiting factor in the literature^19^ , which was no different in this study. The evaluation of consumption from questions specific to F&V, associated with examples of foods, mainly vegetables, may also have influenced the data of quantities consumed. A data validation study showed good accuracy of the report of fruit consumption, but in need of adjustments for vegetable intake^19^ .

This study assessed the food environment of the health service and not solely of the individual, which can be a limiting factor but, also, a differential. The health service analyzed seeks to build health environments and should, therefore, work in conjunction with other public policies of health in the territory. In addition, the study analyzed only aspects of the consumer’s food environment; however, aspects such as type of establishment, density and distance can be significant determinants for individual consumption. Nevertheless, this indicator considers the importance of the consumer environment, as study by the very participants of a qualitative study^29^ .

Results showed that participants of a health promotion program consume the recommended amount of F&V but monotonously, despite the diversity, variety and reasonable quality of F&V in the commercial establishments around the PAS territories. As stated, food monotony reduces the spectrum of nutrients consumed, which can compromise the protective effect of F&V against chronic diseases. Educative actions, therefore, must work beyond the recommended quantities of foods, rescuing the food culture, culinary skills and the importance of the diversified intake of foods, as recommended by the diet guidelines for the Brazilian population.
