# Supplementary material for: Monotony in the consumption of fruits and vegetables and food environment characteristics
Source: Rev Saude Publica. 2019 Aug 23;53:63. doi: 10.11606/S1518-8787.2019053000705 (PMC6707622; doi:10.11606/S1518-8787.2019053000705)
Supplement: Translation

# Translation: Monotonia no consumo de frutas e hortaliças e características do ambiente alimentar

**Authors:** Raquel de Deus Mendonça, Mariana Souza Lopes, Patrícia Pinheiro Freitas, Suellen Fabiane Campos, Mariana Carvalho de Menezes, Aline Cristine Souza Lopes

**Affiliations:** I Universidade Federal de Minas Gerais. Escola de Enfermagem. Departamento de Nutrição. Programa de Pós-Graduação em Enfermagem. Grupo de Pesquisa de Intervenções em Nutrição. Belo Horizonte, MG, Brasil; II Universidade Federal de Minas Gerais. Faculdade de Medicina. Departamento de Medicina Preventiva e Social. Programa de Pós-Graduação em Saúde Pública. Grupo de Pesquisas de Intervenções em Nutrição. Belo Horizonte, MG, Brasil; III Secretaria Municipal de Saúde. Núcleo de Apoio a Saúde da Família. Belo Horizonte, MG, Brasil; IV Fundação Oswaldo Cruz. Escola Nacional de Saúde Pública. Programa de Pós-Graduação de Epidemiologia em Saúde Pública. Grupo de Pesquisas de Intervenções em Nutrição. Rio de Janeiro, RJ, Brasil

**Keywords:** Consumo de Alimentos, Verduras, Frutas, Inquéritos sobre Dietas, Educação Alimentar e Nutricional

## Abstract

**OBJETIVO:**

Analisar a quantidade e a diversidade do consumo de frutas e hortaliças, assim como sua relação com características da aquisição e do ambiente alimentar do consumidor.

**MÉTODOS:**

Estudo da linha de base oriundo de ensaio comunitário controlado e aleatorizado que investigou amostra representativa de serviços da atenção primária à Saúde (Programa Academia da Saúde) de Belo Horizonte, MG. Analisou-se o consumo de frutas e hortaliças em porções/dia, e sua diversidade por Questionário de Frequência Alimentar. Os usuários também foram questionados sobre frequência, local de compra e disponibilidade dos alimentos no domicílio. Para aferir o ambiente alimentar do consumidor, realizou-se auditoria dos estabelecimentos comerciais dentro de um raio de 1,6 km ao redor das unidades amostradas do programa.

**RESULTADOS:**

Investigaram-se 3.414 adultos e idosos (88,1% mulheres) e 336 estabelecimentos comerciais em 18 unidades do Programa Academia da Saúde. O consumo médio de frutas e hortaliças foi adequado [5,4 (DP = 2,1) porções/dia], porém monótono, com ingestão média diária de dois tipos. Nos estabelecimentos auditados, observou-se boa diversidade (77,7% e 85,0%) e variedade (74,5% e 81,4%) de frutas e hortaliças, mas com qualidade inferior de hortaliças (60,4%). Após ajuste por variáveis sociodemográficas, identificou-se que o conhecimento sobre safras de alimentos (p = 0,006), aumento da disponibilidade mensal de frutas nos domicílios (p < 0,001), maior variedade de frutas (p = 0,03) e qualidade das hortaliças (p = 0,05) nos estabelecimentos comerciais poderiam melhorar o consumo quantitativo de frutas e hortaliças, enquanto a maior variedade de frutas (p = 0,008) poderia ampliar a diversidade do consumo.

**CONCLUSÕES:**

O consumo de frutas e hortaliças foi quantitativamente adequado, porém monótono, sendo influenciado pelo ambiente do consumidor. Tais resultados evidenciam a necessidade de aperfeiçoar as ações educativas nos serviços e programas de saúde, além de atuar sobre o ambiente do consumidor, visando promover e manter o consumo adequado e diversificado, conforme preconizado pelas diretrizes brasileiras para a alimentação adequada e saudável.

## INTRODUÇÃO

Ao longo dos séculos, o homem desenvolveu tecnologias para melhorar a disponibilidade e a durabilidade dos alimentos. Passo importante nesse sentido foi a industrialização^1^ , que em pouco tempo permitiu que inúmeros países fossem dominados pelo processamento de alimentos^2^ .

Como consequência da industrialização, o consumo de alimentos *in natura* decresceu^2^ , incluindo as frutas e hortaliças (FH)^3 , 4^ . A inadequação no consumo de FH é um dos dez fatores centrais na determinação da carga global de doenças e caracteriza-se pela quantidade insuficiente ou falta de variedade, com consequente redução do repertório alimentar e a baixa ingestão de nutrientes^5^ .

O consumo inadequado de FH é global e constantemente investigado por estudos e inquéritos populacionais de diversos países^3 , 4 , 6^ . Entretanto, nenhum deles avaliou a diversidade de FH consumida. Nesse contexto, mesmo que as estratégias que visam ampliar o acesso e o consumo de FH sejam prioridade das políticas de saúde no mundo^5^ , elas se limitam a estimular e avaliar o consumo segundo as recomendações (cinco porções/dias). Em geral, não consideram a importância da diversidade na alimentação e pouco abordam o papel do ambiente alimentar sobre o consumo de alimentos, sobretudo em contextos coletivos como os serviços de saúde^10 , 11^ e em países em desenvolvimento.

O ambiente pode tanto favorecer comportamentos insalubres como saudáveis, devido a sua forte interferência sobre as decisões de compra e escolhas dos indivíduos^10^ . Estudos demonstram que uma maior variedade e disponibilidade de alimentos no ambiente do consumidor pode favorecer o seu consumo^13 , 14^ . Um estudo de revisão conduzido nos Estados Unidos apontou que a baixa disponibilidade de FH estava associada à pior qualidade da alimentação^14^ .

Diante do exposto, este artigo objetivou analisar a quantidade e a diversidade do consumo de FH e a sua relação com as características de aquisição e do ambiente alimentar do consumidor.

## MÉTODOS

Trata-se de um estudo que analisou a linha de base (2013/2014) de um ensaio comunitário controlado e aleatorizado em amostra probabilística e representativa de unidades do Programa Academia da Saúde (PAS) de Belo Horizonte, Minas Gerais. A investigação contemplou os usuários do PAS e o ambiente alimentar no entorno de seus territórios. Outros detalhes da pesquisa podem ser vistos em Costa et al.^10^ e Menezes et al.^15^

O PAS foi escolhido como cenário da pesquisa por ser um ponto da Atenção Primária que visa contribuir para a construção de ambientes promotores de saúde. Para isso, conta com infraestrutura, equipamentos e quadro de pessoal qualificado para a orientação de modos saudáveis de vida, incluindo a promoção da alimentação adequada e saudável, e para ações de segurança alimentar e nutricional^16 , 17^ . No município investigado o PAS oferta a prática de exercício físico orientado e ações de promoção da saúde em parceria com o Núcleo de Apoio à Saúde de Família (NASF)^17^ .

O estudo foi conduzido dentro dos padrões exigidos pela Declaração de Helsinque e aprovado pelos comitês de ética em pesquisa da Universidade Federal de Minas Gerais (0537.0.0203.000-11) e da Prefeitura Municipal de Belo Horizonte (0537.0.0203.410-11A). Além disso, foi cadastrado no Registro Brasileiro de Ensaios Clínicos (RBR-9h7ckx), segundo os critérios requeridos pelo *International Committee of Medical Journal Editors* (ICMJE) e a Organização Mundial de Saúde (OMS).

### Amostragem das Unidades do PAS Participantes do Estudo

No processo amostral, foram consideradas 42 unidades do PAS das 50 instaladas no município. Foram excluídas seis unidades localizadas em áreas de baixa vulnerabilidade à saúde por seu reduzido número no município e outras duas pela intensa realização de estudos de intervenção. Dessa forma, foram sorteadas 18 (42,8%) unidades distribuídas pelas nove regiões do município^10^ , representativas do total, com nível de confiança de 95% e erro de 1,4%. Após a coleta de dados, visando verificar a manutenção da representatividade da amostra, foram realizadas análises estatísticas comparando os dados sociodemográficos de todas as unidades (n = 50) com os da amostra (n = 18), com nível de confiança e erro mantidos.

Nas unidades amostradas, todos os participantes foram convidados a participar do estudo (n = 3.763). Desses, 3.414 (90,7%) foram entrevistados, sendo contabilizadas 237 recusas (6,3%) e 112 exclusões (3,0%)^15^ . Os critérios de inclusão eram: ter pelo menos 20 anos de idade e ter participado regularmente das atividades do PAS no mês anterior ao início da coleta de dados. Os critérios de exclusão foram ser gestante e ter comprometimento cognitivo que impossibilitasse responder ao questionário. Os dados da participação nas atividades foram coletados por meio do registro de frequência diária realizado pelo serviço, enquanto o comprometimento cognitivo foi constatado a partir da avaliação inicial do usuário no seu ingresso no PAS e da avaliação feita pela equipe de pesquisa^15 , 17^ .

Para caracterizar o ambiente alimentar, foram investigados os estabelecimentos que comercializavam FH e feiras livres contidas em um *buffer* com raio de 1.600 metros ao redor de cada unidade do PAS amostrada. Esta distância foi escolhida por ser factível para realizar as compras sem necessidade de transporte motorizado. Os dados dos estabelecimentos e feiras livres foram disponibilizados pelo município e adicionados àqueles encontrados espontaneamente no território das unidades do PAS durante a visita aos estabelecimentos registrados^10^ .

A coleta de dados foi realizada por acadêmicos de nutrição e profissionais de saúde treinados semestralmente para o uso dos instrumentos e a condução de entrevistas, sob a orientação de supervisor de campo e do pesquisador principal. Os dados passaram por análise de consistência antes e após a tabulação.

### Avaliação Individual

As informações dos indivíduos foram obtidas por questionário submetido a estudo piloto e aplicado face a face por entrevistadores treinados. Foi construído a partir de estudos nacionais e experiência pregressa do grupo de pesquisa^8 , 15 , 18 , 19^ e contemplou características sociodemográficas e econômicas, aspectos de saúde, perfil de aquisição, estado nutricional e consumo de FH *.*

Para registrar o consumo de FH, utilizou-se o Questionário Breve de Avaliação do Consumo de Frutas e Hortaliças (QBreve-FH), validado para a população de estudo^19^ , que investigou a frequência (1–2 dias/semana, 3–4 dias/semana, 5–6 dias/semana, diariamente – inclusive sábado e domingo, quase nunca/nunca) e o número de porções consumidas. Para verificar a diversidade do consumo, foi aplicado o Questionário de Frequência Alimentar (QFA) específico, relativo aos últimos seis meses, incluindo 14 frutas e 22 hortaliças. A diversidade foi avaliada pelo consumo diário das FH analisadas^19^ . Para caracterizar o perfil de aquisição de FH, utilizaram-se as variáveis relativas ao conhecimento sobre o conceito de safra de alimentos e disponibilidade de FH no domicílio, além de frequência e tipo de estabelecimento escolhido para a compra de FH^15^ .

As características sociodemográficas e econômicas investigadas foram sexo, idade, escolaridade (anos), estado civil (união estável, separado, solteiro ou viúvo) e ocupação (do lar, aposentado, empregado ou desempregado). Bens materiais na residência e o grau de instrução do chefe da família foram utilizados para a classificação econômica dos participantes em cinco classes, de A (a mais rica) até a E (a mais pobre), segundo o Critério de Classificação Econômica Brasil^20^ .

Aspectos de saúde foram avaliados pelo autorrelato de diagnóstico médico de diabetes, hipertensão arterial e dislipidemia, assim como pela percepção de saúde (muito boa, boa, regular, ruim ou muito ruim). Além disso, coletaram-se informações sobre a frequência (dias) e duração (minutos) da prática de exercícios físicos e sobre o tempo de participação do usuário no PAS (diferença entre as datas de coleta dos dados e do ingresso do usuário no programa).

Para a avaliação do estado nutricional, foram utilizadas as medidas antropométricas de peso e altura, com base nos quais foi calculado o índice de massa corporal (IMC), classificado conforme critérios definidos pela OMS^21^ . O peso foi aferido por medida única em balança digital da marca Marte^®^, com capacidade para 180 kg e precisão de 100 g. A estatura também foi verificada por medida única em estadiômetro portátil, marca Alturexata^®^, com capacidade para 220 cm, adotando as técnicas preconizadas pelo Ministério da Saúde^22^ .

### Avaliação do Ambiente Alimentar

Realizou-se visita a lojas de conveniência, mercado municipal, sacolões de rede privada e subsidiados pela prefeitura, mercados locais ou de bairro, supermercados de grandes redes, hipermercados, supermercados de atacarejo e padarias^23^ . Foram verificadas as seguintes características do ambiente do consumidor para as FH: diversidade, variedade e qualidade. Para isso, utilizaram-se instrumentos desenvolvidos e validados para o contexto brasileiro^24^ , que foram adaptados incluindo os 10 tipos mais adquiridos de frutas e hortaliças em Belo Horizonte (exceto frutas e hortaliças enlatadas ou congeladas e tubérculos e raízes), conforme a Pesquisa de Orçamentos Familiares 2008–2009^8^ .

A diversidade foi avaliada pelo o número de FH disponíveis no estabelecimento, enquanto a variedade foi pelo número de diferentes tipos de FH de uma mesma espécie (por exemplo, maçã verde, maçã gala ou maçã fuji). A qualidade foi classificada em boa ou ruim a partir de avaliação subjetiva, considerando a presença de manchas e machucados ou aparência de “passado” ou maduro demais^24^ .

### Análise dos Dados

O consumo de FH foi analisado em quintis de porções diárias consumidas e descrito de maneira agregada segundo as unidades do PAS investigadas. Já as características do ambiente alimentar foram analisadas segundo as unidades do PAS.

Para verificar a associação entre as características dos participantes e os quintis de consumo de FH, utilizou-se o teste estatístico de qui-quadrado de tendência linear para as variáveis categóricas e análise de variância (Anova) para as variáveis contínuas. A regressão linear multinível foi utilizada para avaliar se os indicadores do perfil de aquisição de alimentos (nível individual) e do ambiente alimentar do consumidor (diversidade, variedade e qualidade de frutas e hortaliças, totalizando seis variáveis) explicavam as variações no consumo quantitativo (porção/dia) e qualitativo (diversidade diária) de FH. Essa análise permite considerar a estrutura hierárquica do nível de agregação dos dados, além da inserção de variáveis individuais e contextuais. Assim, o consumo individual (porções diária e diversidade) de FH consistiu nos dois desfechos analisados, com os participantes (nível 1) aninhados de acordo com as unidades do PAS (nível 2).

Além do perfil de aquisição de alimentos no nível individual, foram adicionadas variáveis sociodemográficas para ajustamento do modelo. A construção do modelo se deu de forma hierárquica, sendo primeiro construído o modelo nulo (com inclusão apenas do intercepto randômico), seguido da inserção das variáveis individuais, e por fim das variáveis do ambiente. Os modelos finais mostraram as variáveis que apresentaram maior associação (menor valor p e maior porcentagem de variabilidade explicada) com os desfechos porções de FH e diversidade consumida, após ajuste pelas variáveis individuais. Destaca-se que esses são modelos com intercepto aleatório, que não apresentam esse padrão para os coeficientes (de efeitos fixos).

O coeficiente de correlação intraclasse (ICC) foi quantificado para analisar a variabilidade dentro e entre os territórios, fornecendo a proporção da variabilidade total, que depende das diferenças entre os territórios. A porcentagem de variação proporcional na variância (PVP) foi calculada entre o modelo nulo e o modelo final, visando analisar o quanto as covariáveis explicaram a variação nos desfechos entre os territórios.

Todas as variáveis foram analisadas no programa Stata/SE versão 14.0 ( *Stata Corp., College Station* , TX, USA), e a significância estatística foi estabelecida em 5%.

## RESULTADOS

Foram entrevistados 3.414 indivíduos e auditados 336 estabelecimentos comerciais presentes nos territórios de 18 unidades do PAS. A maioria dos participantes eram mulheres (88,1%) e adultos de meia idade [56,7 (SD = 11,7) anos], com consumo médio relatado de 5,4 (SD = 2,1) porções de FH de dois tipos diferentes por dia ( Tabela 1 ).

**Tabela 1:** Características dos participantes segundo quintil de consumo de frutas e hortaliças. Belo Horizonte, MG, 2013–2014.

Variável	Total	Quintil do consumo total de frutas e hortaliças					p*

		Q1	Q2	Q3	Q4	Q5	
Frutas e hortaliças (porções/dia)	5,4 (2,1)	3,0 (0,6)	4,3 (0,3)	5,2 (0,2)	6,2 (0,3)	8,5 (1,9)	< 0,001
Idade (anos)	56,7 (11,7)	54,7 (12,6)	56,2 (11,8)	56,8 (11,3)	57,7 (11,3)	58,6 (11,1)	< 0,001
Sexo (%)							0,001
Feminino	88,1	89,5	90,1	88,0	86,9	84,5	
Masculino	11,9	10,5	9,9	11,0	13,1	15,5	
Anos de estudo	7,2 (4,1)	7,1 (4,2)	7,1 (4,0)	7,5 (4,1)	7,2 (4,0)	7,3 (4,2)	0,39
Estado civil (%)							0,71
União estável	61,6	60,8	62,9	62,5	61,0	61,0	
Separado	8,3	8,2	8,3	7,9	7,8	9,2	
Solteiro	14,1	15,9	13,6	13,1	13,9	13,7	
Viúvo	16,0	15,1	15,2	16,5	17,3	16,1	
Ocupação (%)							0,02
Do lar	28,7	31,1	27,8	29,3	29,6	25,5	
Aposentado	36,7	30,4	33,9	36,3	40,4	43,8	
Desempregado	2,0	2,9	1,9	2,1	1,8	1,2	
Empregado	32,6	35,6	36,4	32,4	28,3	29,5	
Classe econômica (%)							0,83
A/B	29,5	28,7	30,0	30,1	29,7	29,2	
C	55,0	53,3	55,6	57,3	56,0	53,1	
D/E	15,5	18,0	14,4	12,6	14,3	17,7	
Tempo no PAS (meses)	19,8 (15,3)	18,8 (15,1)	19,5 (14,7)	20,0 (15,7)	20,5 (15,7)	20,4 (15,3)	0,21
IMC (kg/m^2^)	27,9 (4,8)	27,6 (4,8)	27,6 (4,8)	28,2 (4,7)	27,9 (5,0)	28,0 (5,1)	0,06
Hábito de fumar (%)	5,7	6,1	4,3	7,2	5,2	5,6	0,99
Doenças crônicas (%)							
Diabetes	16,9	12,5	13,9	18,3	19,9	21,0	< 0,001
Hipertensão arterial	53,2	47,8	51,8	54,4	57,1	55,9	< 0,001
Dislipidemia	44,1	41,2	42,5	46,1	46,5	44,8	0,62
Percepção de saúde (%)							0,48
Muito boa/boa	71,7	71,4	73,4	70,7	73,7	69,6	
Regular/Ruim/Muito ruim	28,3	28,6	26,6	29,3	26,3	30,4	
Prática de exercícios físicos (minutos/semana)	209,6 (79,9)	201,9 (63,4)	206,8 (71,7)	208,6 (86,9)	218,3 (89,8)	213,0 (84,8)	0,004
Diversidade diária (tipos)							
Frutas	1,5 (1,4)	0,8 (1,0)	1,2 (1,3)	1,6 (1,4)	1,7 (1,4)	1,9 (1,5)	< 0,001
Hortaliças	0,9 (1,4)	0,7 (1,2)	0,8 (1,3)	0,9 (1,4)	0,9 (1,2)	1,1 (1,6)	< 0,001
Frutas e hortaliças	2,3 (2,1)	1,6 (1,7)	2,1 (2,0)	2,6 (2,3)	2,6 (2,2)	3,1 (2,4)	< 0,001

PAS: Programa Academia da Saúde; IMC: índice de massa corporal

Valores apresentados como % ou como média (desvio-padrão).

* Qui-quadrado de tendência linear (variáveis categóricas) e análise de variância (Anova; variáveis contínuas), segundo quintil de consumo de frutas e hortaliças.

Participantes no último quintil de consumo de FH [8,5 (SD = 1,9) porções/dia], comparados com os do primeiro [3,0 (SD = 0,6) porções/dia], apresentavam idade mais avançada, maior proporção de aposentados, de portadores de alguma doença crônica e de indivíduos mais ativos fisicamente. As mulheres relataram maior consumo de FH, que reduziu com o aumento dos quintis de FH, situação oposta à dos homens ( Tabela 1 ).

Indivíduos no último quintil de consumo de FH relataram ingerir maior diversidade desses alimentos que os do primeiro quintil ( Tabela 1 ). As principais frutas consumidas foram banana, laranja e maçã, e as hortaliças foram tomate, alface e cenoura ( Figura ). Pessoas com maior consumo de FH relataram maior conhecimento sobre a safra dos alimentos, realizavam suas compras com maior frequência (aproximadamente uma vez por semana) e apresentavam maior disponibilidade de FH no domicílio ( Tabela 2 ).

**Figura:** Principais frutas e hortaliças consumidas diariamente. Belo Horizonte, MG, 2013–2014.

**Tabela 2:** Perfil de aquisição de alimentos segundo quintil de consumo de frutas e hortaliças. Belo Horizonte, MG, 2013–2014.

Variável	Total	Quintil do consumo total de frutas e hortaliças					p*

		1	2	3	4	5	
Conhecimento sobre safras (%)	75,0	70,8	71,7	78,7	76,4	77,9	< 0,001
Estabelecimento onde compra frutas e hortaliças (%)							
Supermercados	40,5	41,6	39,4	39,7	41,3	40,4	0,91
Sacolão subsidiado	40,8	38,6	39,1	43,5	39,2	43,7	0,07
Sacolão privado	80,5	82,0	80,9	80,0	81,1	78,5	0,14
Feiras livres	81,2	80,0	82,1	82,4	80,6	81,0	0,89
Mercado municipal	23,7	25,5	23,0	23,5	25,3	21,4	0,23
Horta ou doação	22,8	22,1	22,9	21,2	24,7	23,5	0,37
Frequência de aquisição de frutas (%)							< 0,001
3 vezes ou mais/semana	8,6	6,5	8,4	8,4	8,8	11,3	
1–2 vezes/semana	77,9	73,8	79,0	76,6	80,7	79,7	
3 vezes ou menos/mês	13,5	19,7	12,6	15,0	10,5	9,0	
Frequência de aquisição de hortaliças (%)							< 0,001
3 vezes ou mais/semana	12,9	10,6	12,3	11,9	12,9	16,8	
1 a 2 vezes/semana	76,1	73,1	76,9	76,9	77,9	76,0	
3 vezes ou menos/mês	11,0	16,3	10,8	11,2	9,2	7,2	
Disponibilidade mensal (dias)							
Frutas	27,1 (6,6)	25,5 (8,3)	27,1 (6,5)	27,2 (6,3)	28,2 (5,3)	27,9 (5,8)	< 0,001
Hortaliças	28,1 (5,5)	27,4 (6,7)	27,9 (5,7)	28,1 (5,6)	29,0 (4,0)	28,6 (5,0)	< 0,001

Valores apresentados como % ou como média (desvio-padrão).

* Qui-quadrado de tendência linear (variáveis categóricas) e análise de variância (Anova; variáveis contínuas), segundo quintil de consumo de frutas e hortaliças.

As características do ambiente alimentar do consumidor segundo as unidades do PAS investigadas estão apresentadas na Tabela 3 . A maioria apresentou variedade e diversidade adequada, com percentuais acima de 70%, mas qualidade inferior (< 60%).

**Tabela 3:** Consumo de frutas e hortaliças e ambiente alimentar do consumidor no Programa Academia da Saúde. Belo Horizonte, MG, 2013–2014.

Região	Unidade do PAS	Adequação (%)					

		Variedade de frutas	Variedade de hortaliças	Diversidade de frutas	Diversidade de hortaliças	Qualidade de frutas	Qualidade de hortaliças
1	1	70,0	70,0	70,0	80,0	85,7	87,5
	2	90,0	90,0	90,0	90,0	100,0	77,8
2	3	77,1	79,2	77,1	72,2	94,1	90,9
	4	83,3	83,3	91,7	75,0	90,0	100,0
3	5	63,6	81,8	75,8	93,9	54,1	38,7
	6	68,7	80,6	80,6	93,5	65,2	37,9
4	7	70,6	88,2	76,5	88,5	54,5	33,3
	8	43,7	62,5	50,0	88,2	73,3	42,8
5	9	83,3	83,3	83,3	68,5	73,3	56,2
	10	70,0	70,0	70,0	83,3	57,1	25,0
6	11	81,8	63,6	81,8	70,0	71,4	50,0
	12	77,8	55,6	77,8	81,8	60,0	62,5
7	13	88,2	100,0	88,2	77,8	80,0	70,6
	14	81,2	95,4	90,9	100,0	78,9	77,2
8	15	70,0	90,0	70,0	95,4	50,0	22,2
	16	71,4	73,3	80,0	90,0	81,2	33,3
9	17	87,1	80,6	87,1	80,0	96,0	82,1
	18	87,7	81,2	87,5	87,5	84,6	73,3

Total		75,4	80,6	79,8	84,2	75,5	60,4

PAS: Programa Academia da Saúde; FH: frutas e hortaliças

Variedade: número de diferentes tipos de FH de uma mesma espécie no estabelecimento.

Diversidade: número de FH disponíveis no estabelecimento.

Na análise multinível, após ajuste por variáveis sociodemográficas, verificou-se que o conhecimento sobre safras de alimentos e o aumento da disponibilidade mensal no domicílio e da frequência de compra de frutas poderia aumentar o consumo (porções/dia) de FH ( Tabela 4 ). Em relação ao ambiente, um maior consumo (porções/dia) foi associado a estabelecimentos comerciais com maior variedade de frutas e qualidade de hortaliças, independentemente das variáveis individuais. A variância intragrupo e o ICC reduziram progressivamente até o modelo final apresentado (modelo nulo: σ^2^= 2,04, ICC = 0,011; modelo nível 1: σ^2^= 2,02, ICC = 0,009; modelo níveis 1 e 2: σ^2^= 2,01, ICC = 0,004), indicando que as covariáveis inseridas auxiliaram a explicar o consumo de FH. Alinhado a esse resultado, o cálculo da PVP demonstrou que as covariáveis do modelo explicaram 34,7% da variável do consumo quantitativo de FH.

**Tabela 4:** Modelos de regressão linear multinível para consumo de frutas e hortaliças quantitativo (em porções) e qualitativo (variedade de consumo) como variáveis desfecho. Belo Horizonte, MG, 2013–2014.

Variáveis explicativas	B	Erro-padrão	IC95%	p
Modelo com variável desfecho consumo de frutas e hortaliças (porções)				

Variáveis individuais – nível 1				
Conhecimento sobre safras de alimentos^a^	0,27	0,09	0,08–0,46	0,006
Compra frutas 3 vezes ou mais/semana	0,58	0,18	0,22–0,94	0,002
Compra frutas 1 a 2 vezes/semana	0,33	0,13	0,07–0,58	0,012
Disponibilidade mensal de frutas (dias)	0,03	0,01	0,02–0,05	< 0,001
Variáveis de ajuste				
Idade (anos)	0,02	0,00	0,01–0,02	< 0,001
Sexo	0,52	0,13	0,27–0,77	< 0,001
Anos de estudo	0,01	0,01	-0,01–0,03	0,56
Variáveis do ambiente – nível 2				
Variedade de frutas	1,07	0,48	0,13–2,01	0,03
Qualidade de hortaliças^b^	1,59	0,83	-0,03–3,21	0,05
Efeito aleatório				
Variância entre grupos (τ00)	0,14	0,06	0,06–0,33	-
Variância intragrupo (σ^2^)	2,02	0,03	1,96–2,07	-

Modelo com variável desfecho diversidade no consumo de frutas e hortaliças				

Variáveis individuais – nível 1				
Variáveis de ajuste				
Idade (anos)	0,00	0,00	-0,01–0,01	0,58
Sexo	0,05	0,12	-0,20–0,30	0,70
Anos de estudo	0,00	0,01	-0,02–0,02	0,79
Variáveis do ambiente – nível 2				
Diversidade de frutas	0,27	0,10	0,07–0,46	0,008
Qualidade de hortaliças^b^	0,32	020	0,07–0,70	0,11
Efeito aleatório				
Variância entre grupos (τ00)	0,31	0,08	0,19–0,53	-
Variância intragrupo (σ^2^)	2,02	0,03	1,97–2,08	-

B: Coeficiente B

^a^Conhece safras de alimentos (não ou sim).

^b^Qualidade de hortaliças (ruim ou boa).

As variáveis do perfil de aquisição de alimentos investigadas não se associaram à diversidade de FH consumidos, mas as variáveis de oferta dos territórios, sim. As covariáveis inseridas no modelo ajudaram a explicar 28,3% da variedade do consumo de FH.

## DISCUSSÃO

Os participantes desse programa de promoção da saúde, parte da atenção primária brasileira, relataram consumo adequado de FH (média diária de cinco porções). Entretanto, ao avaliar o consumo qualitativamente, verificou-se monotonia alimentar, com uma média de apenas dois tipos diferentes de FH ingeridos por dia. O perfil de aquisição de FH interferiu no consumo, com destaque para o conhecimento sobre safras de alimentos e frequência de compra e disponibilidade domiciliar de frutas. O ambiente alimentar do consumidor, por sua vez, demonstrou ser importante tanto para o consumo quantitativo (porções/dia) como para o qualitativo (diversidade do consumo).

Essa quantidade foi surpreendentemente superior aos valores nacionais e internacionais^3 , 4 , 6 , 7^ . Na Inglaterra (2011/2012), 70% dos adultos e 59% dos idosos não consumiam a quantidade recomendada de FH^3^ . Nos Estados Unidos, o consumo diário médio de tais alimentos é de 2,7 porções ao dia^4^ . Na América Latina, em países como Argentina (2013) e Chile (2009/2010), 5,0% e 84,3% da população, respectivamente, não apresentavam consumo adequado^6 , 7^ . Pesquisas realizadas no Brasil (de 1987 a 2009) evidenciam declínio das quantidades médias adquiridas^25^ e do consumo de FH^26^ , estimando que menos de 10,0% da população apresentava consumo adequado^8^ .

A ingestão encontrada neste estudo também foi superior à identificada em outro trabalho realizado no PAS. Ao analisar os ingressantes de um triênio (2008 a 2010) no PAS, Costa et al.^27^ verificaram inadequação de 75,3% no consumo de FH. Parte da diferença dos resultados pode ser atribuída ao tempo de participação do usuário no serviço de saúde; a média deste estudo era de 20 meses. A participação em serviço de promoção da saúde, ainda que voltado para a prática de exercícios físicos, parece potencializar a adoção de outros hábitos saudáveis de vida, como a alimentação saudável, além de melhorar o acesso a conhecimentos como os referentes à safra dos alimentos^16^ .

Neste estudo, portanto, observou-se o potencial desse serviço de promoção da saúde para reverter a deterioração quantitativa do consumo de FH *.* Tal resultado revela um possível caminho para os governos promoverem mudanças positivas nesse aspecto. Entretanto, ele também aponta o desafio da monotonia alimentar e a iminente necessidade de sua discussão no contexto das recomendações e das ações educativas, resgatando e elucidando a mensagem da OMS de que o consumo adequado consta da ingestão regular, diversificada e quantitativamente recomendada de frutas e hortaliças^5^ .

Há mais de 200 tipos de frutas e hortaliças presentes em diferentes culturas, que se diferenciam pela densidade calórica, quantidade de água e diversidade de micronutrientes, os quais acarretam benefícios para a saúde e proteção contra doenças^5 , 28^ . A diversidade alimentar insuficiente pode prejudicar a manutenção de hábitos alimentares saudáveis em longo prazo, resultando na redução do repertório de alimentos da população e no menor teor nutricional da alimentação.

A baixa diversidade no consumo de FH pode também se relacionar ao menor preço, tempo e complexidade exigida para o preparo dos alimentos, assim como a uma tentativa de superar obstáculos como baixa qualidade das FH. Tais questões se tornam ainda mais relevantes ao considerar que foram estudadas áreas vulneráveis, com média de renda abaixo da população brasileira^23 , 29^ .

Um estudo prévio sobre estabelecimentos comerciais no território do PAS de Belo Horizonte demonstrou que em algumas unidades havia acesso limitado a estabelecimentos com adequada disponibilidade e variedade de FH^10^ . O ambiente alimentar deve apoiar escolhas saudáveis, ofertando opções amplas de alimentos acessíveis, variados e de qualidade. Este estudo reforça essas informações ao sugerir que as variáveis do ambiente alimentar do consumidor (variedade e qualidade) foram importantes, além de questões relativas à aquisição de FH. Em concordância com os resultados, em um trabalho realizado no Brasil, o relato de mulheres sobre a aquisição de alimentos revelou que a variedade de FH nos estabelecimentos impactava a aquisição de alimentos saudáveis em detrimento dos alimentos ultraprocessados^30^ .

Nossos resultados enfatizam a importância de promover a alimentação adequada, diversificada e saudável na atenção primária à saúde, tendo em vista a sua potencialidade de incentivar modos saudáveis de viver. Entretanto, é preciso avançar para além da adequação quantitativa e dos fatores associados, estudando também a diversidade consumida^5 , 28^ . Além disso, pouco ainda se sabe sobre a influência do ambiente do consumidor na alimentação, situação que evidencia como ainda se trata a alimentação fora da complexidade na qual está imersa.

Sugere-se a realização de mais estudos, visando compreender melhor a relação entre consumo, monotonia e ambiente alimentar (oferta e demanda) e buscando responder a questionamentos como: o ambiente restrito gera a monotonia alimentar ou é a monotonia alimentar (demanda pouco diversificada) que leva à oferta precária de alimentos no território?

Esses resultados, apesar de inéditos e relevantes, requerem cautela quanto à validade externa. Os participantes são frequentadores de um serviço direcionado para a promoção da saúde, com enfoque na prática de exercício físico, e, portanto, mais conscientes quanto à sua saúde, o que os diferencia da população em geral^9^ . Entretanto, os resultados mostram a importância de trabalhar em outros cenários de pesquisa, bem como de entender as ações ofertadas nos serviços de saúde como possíveis caminhos para enfrentar os desafios enfrentados pelo setor. A dificuldade para obter medidas fidedignas do consumo de alimentos tem sido apontada como importante limitador na literatura^19^ , o que não foi diferente neste estudo. A avaliação do consumo com perguntas específicas para FH associadas com exemplos de alimentos, em especial de hortaliças, também pode ter influenciado os dados de quantidades consumidas. Um estudo de validação dos dados mostrou boa precisão do relato do consumo de frutas, mas com necessidades de ajustes para a ingestão de hortaliças^19^ .

Este trabalho avaliou o ambiente alimentar do serviço de saúde e não exclusivamente do indivíduo, o que pode ser um limitante, mas também um diferencial. O serviço de saúde analisado busca a construção de ambientes saudáveis, devendo, portanto, trabalhar de maneira integrada com outras políticas públicas de saúde do território. Além disso, o estudo analisou apenas aspectos do ambiente alimentar do consumidor, mas aspectos como tipo de estabelecimento, densidade e distância podem ser determinantes consideráveis para o consumo individual. Contudo, pondera a importância dos aspectos do ambiente do consumidor, como afirmado pelos próprios participantes em estudo qualitativo^29^ .

Os resultados revelam que participantes de um programa de promoção da saúde consomem FH na quantidade recomendada, mas de forma monótona, apesar da diversidade, variedade e qualidade razoável de FH nos estabelecimentos comerciais no entorno dos territórios do PAS. Como dito, a monotonia alimentar reduz o espectro de nutrientes consumidos, podendo comprometer o efeito de proteção das FH contra doenças crônicas. Ações educativas, portanto, devem trabalhar para além das recomendações de quantidades de alimentos, resgatando a cultura alimentar, habilidades culinárias e a importância do consumo diversificado de alimentos, conforme preconizado pelas diretrizes alimentares para a população brasileira.
